# Multi-Spectroscopic and Theoretical Analysis on the Interaction between Human Serum Albumin and a Capsaicin Derivative—RPF101

**DOI:** 10.3390/biom8030078

**Published:** 2018-08-23

**Authors:** Otávio Augusto Chaves, Maurício Temotheo Tavares, Micael Rodrigues Cunha, Roberto Parise-Filho, Carlos Maurício R. Sant’Anna, José Carlos Netto-Ferreira

**Affiliations:** 1 Institute of Chemistry, Universidade Federal Rural do Rio de Janeiro, BR-465 Km 7, 23970-000 Seropédica-RJ, Brazil; otavioaugustochaves@gmail.com (O.A.C.); santana@ufrrj.br (C.M.R.S.); 2 SENAI Innovation Institute for Green Chemistry, Rua Morais e Silva N° 53, Maracanã, 20271030 Rio de Janeiro-RJ, Brazil; 3 Department of Pharmacy, University of São Paulo, Prof. Lineu Prestes Avenue, 580, Bl.13, 05508-900 Butanta, São Paulo-SP, Brazil; mauricio.tavares@usp.br (M.T.T.); micaelrc@usp.br (M.R.C.); roberto.parise@usp.br (R.P.-F.); 4 National Institute of Metrology, Quality and Technology, 25250-020 Duque de Caxias-RJ, Brazil

**Keywords:** human serum albumin, RPF101, capsaicin, multi-spectroscopy, molecular docking

## Abstract

The interaction between the main carrier of endogenous and exogenous compounds in the human bloodstream (human serum albumin, HSA) and a potential anticancer compound (the capsaicin analogue **RPF101**) was investigated by spectroscopic techniques (circular dichroism, steady-state, time-resolved, and synchronous fluorescence), zeta potential, and computational method (molecular docking). Steady-state and time-resolved fluorescence experiments indicated an association in the ground state between HSA:**RPF101**. The interaction is moderate, spontaneous (Δ*G*° < 0), and entropically driven (Δ*S*° = 0.573 ± 0.069 kJ/molK). This association does not perturb significantly the potential surface of the protein, as well as the secondary structure of the albumin and the microenvironment around tyrosine and tryptophan residues. Competitive binding studies indicated Sudlow’s site I as the main protein pocket and molecular docking results suggested hydrogen bonding and hydrophobic interactions as the main binding forces.

## 1. Introduction

**Figure 1:**
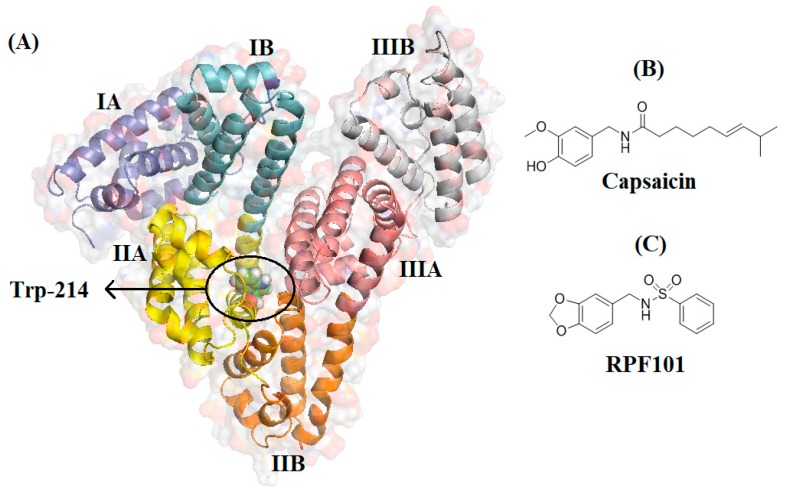
(**A**) Human serum albumin (HSA) structure as cartoon representation and tryptophan-214 (Trp-214) residue as green stick. (**B**,**C**) Chemical structure of capsaicin and its analogue **RPF101**.

Capsaicin is a major component of red pepper, being reported as a potent anticancer compound [1]. However, due to its intrinsic pungent effect through transient receptors potential vanilloid type 1 (TRPV1) [2], to date the therapeutic use of capsaicin is restricted to pain relief by topical application [3]. Using strategies of ligand-based drug design, Tavares et al. [4] and Damião et al. [5] developed a wide number of capsaicin derivatives aiming to enhance their anticancer profile [6,7]. Interestingly, among the active compounds, *N*-(benzo[*d*][1,3]dioxol-5-ylmethyl) benzenesulfonamide (**RPF101**, Figure 1) was revealed as a prominent cytotoxic compound, being active against five different breast and skin cancer cell lines, with no associated pungency in vivo [4]. **RF101** can induce arrest of the cell cycle at the G2/M phase through a disruption of the microtubule network. Furthermore, it can cause cellular morphologic changes characteristic of apoptosis and a decrease of mitochondrial membrane potential (Δψm) [4].

Serum albumin (SA) is the most abundant protein in mammalian blood plasma, being synthesized in the liver, where it is produced at a rate of approximately 0.7 mg/h for every gram of liver (i.e., 10–15 g daily) [8]. Due to its high concentration and to the presence of multiple binding pockets, SA is a major transporter of endogenous compounds, including fatty acids, hormones, and metal ions [9]. Human serum albumin (HSA) is the most abundant protein that is present in the human circulatory system (35–50 g/L), having an average half-life of 19 days. The three-dimensional structure of HSA has been elucidated by X-ray analysis and revealed a non-glycosylated heart shaped molecule (66.5 kDa), with dimensions of 80 Å × 80 Å × 80 Å × 30 Å (Figure 1) [10,11]. Human serum albumin structure is composed by three structurally similar domains I–III, each one consisting of two subdomains (A and B), stabilized by 17 disulfide bridges [12]. Human serum albumin is an acidic, very soluble protein that is extremely robust: it is stable in the pH range of 4–9, soluble in 40% ethanol, and can be heated at 60 °C for up to 10 h without deleterious effects. These properties as well as its preferential uptake in tumor and inflamed tissue, ready availability, biodegradability, and the lack of toxicity and immunogenicity make it an ideal candidate for drug delivery [13]. In addition, as most drugs have a poor pharmacokinetic profile and are generally non-specifically distributed in the human body, sometimes causing serious side effects, the development of drug delivery systems intended to specifically target a tumor site becomes a very important issue. When considering that **RPF101** is a promising anticancer agent and that albumin is the major component in passive and active tumor targeting in the drug delivery process, the study of the interaction between HSA and **RPF101** is of extreme relevancy [14,15].

The present work reports a study of the interaction between HSA and the capsaicin derivative **RPF101** using multi-spectroscopic techniques (circular dichroism, steady state, time-resolved, and synchronous fluorescence), zeta potential, and theoretical calculations (molecular docking). Each of the techniques that are employed in this work makes a significant contribution to the understanding of the binding process. The steady-state fluorescence technique indicates the characteristics of the photophysical process that is associated with the HSA:ligand interaction, as well as on the thermodynamic parameters involved in this interaction. On the other hand, time resolved fluorescence is one of the most sensitive spectroscopic techniques and it allows for confirming which main mechanism is operating in the fluorescence quenching process, whereas the structural perturbation that occurs in the microenvironment around the amino acid residues Tyr (tyrosine) and Trp (tryptophan) can be investigated using the synchronous fluorescence technique. Additional information on both the secondary structure and the albumin surface perturbation after ligand binding can be obtained while using the circular dichroism and zeta potential techniques. Finally, molecular modeling may suggest the major binding forces and amino acid residues involved in the interaction between HSA and **RPF101**. These results will offer a comprehension on the interaction mechanism between HSA—the main carrier of endogenous and exogenous molecules—and the natural product analogue **RPF101**, contributing to an understanding of distribution and transport involved in the behavior of the cytotoxic compound **RPF101**—one of the main steps in drug development [16].

## 2. Materials and Methods

### 2.1. Chemicals

Commercially available HSA, warfarin, ibuprofen, digitoxin, and phosphate-buffered saline (PBS) (pH = 7.4) were purchased from Sigma-Aldrich (São Paulo, SP, Brazil). Phosphate-buffered saline solution was obtained by dissolving one tablet in 200 mL of deionized water, yielding 1.00 × 10^−2^ M of phosphate buffer containing 2.70 × 10^−3^ M potassium chloride and 1.37 × 10^−1^ M sodium chloride—pH 7.4 at 298 K. Water used in all the experiments was millipore grade. Methanol (spectroscopic grade) was obtained from Vetec Quimica Fina Ltd. (Rio de Janeiro, RJ, Brazil). The compound **RPF101** was synthesized following the procedure that was described in the literature [4]. Briefly, triethylamine and dimethylformamide were added to a dichloromethane solution of piperonylamine. Then benzenesulfonyl chloride (5.0 mmol) was added dropwise under nitrogen atmosphere and stirred for 24 h at room temperature. The organic layer was thoroughly washed, dried over MgSO_4_, and the solvent was removed under high vacuum. The product **RPF101** was obtained after recrystalization from hot hexane:dichloromethane, resulting in a white solid (80.2% yield) with a melting point (m.p.) = 77.1–77.6 °C [4].

### 2.2. Steady-State Fluorescence Measurements

Steady-state fluorescence was measured on a Jasco J-815 fluorimeter in a quartz cell (1 cm optical path), employing a thermostated cuvette holder Jasco PFD-425S15F (JascoEaston, MD, USA). All spectra were recorded with appropriate background corrections, with number of averaging of three scans for each recorded. In order to compensate the inner filter effect (see Figure S1 in the Supplementary Material), the steady-state fluorescence intensity values for the association HSA:**RPF101** was corrected through the absorption of **RPF101** at excitation and emission wavelengths, according to Equation (1) [17]:

(1) $$F_{cor}=F_{obs}10^{\left[(A_{ex}+A_{em})/2\right]}$$

where, *F_cor_* and *F_obs_* are the corrected and observed steady-state fluorescence intensity values, respectively. *A_ex_* and *A_em_* are the experimental absorbance value at the excitation (molar extinction coefficient at 280 nm−ε = 10,364 M^−1^ cm^−1^) and emission wavelength (molar extinction coefficient at 340 nm−ε = 808.75 M^−1^ cm^−1^), respectively.

The steady-state fluorescence spectra were measured in the 290–450 nm range, at 296, 303, and 310 K, with excitation wavelength at 280 nm. To a 3.0 mL solution containing an appropriate concentration of HSA (1.00 × 10^−5^ M), successive aliquots from a stock solution of **RPF101** (1.00 × 10^−3^ M in methanol) were added, with final concentrations of 0.17; 0.33; 0.50; 0.66; 0.83; 0.99; 1.15; 1.32 × 10^−5^ M. The addition was done manually by using a micro syringe. To investigate a possible perturbation on the steady-state fluorescence and circular dichroism spectra for HSA by adding methanol (solvent used for **RPF101**), these spectra were recorded without and in the presence of 40 µL of this solvent. No significant effect was observed in both cases (see Figure S2 in the Supplementary Material).

### 2.3. Time-Resolved Fluorescence Measurements

Time-resolved fluorescence measurements were performed in a model FL920 CD Edinburgh Instruments fluorimeter (Edinburgh, UK) equipped with an electrically pumped laser (EPL) (λ_exc_ = 280 ± 10 nm; pulse of 850 ps with energy of 1.8 µW/pulse; monitoring emission at 340 nm). The time range was fixed for 80 ns, with channels 512 (time/ch = 0.09766 ns) and peak counts of 700 counts. Fluorescence decay was obtained for the free HSA solution (1.00 × 10^−5^ M in PBS) and for a HSA solution containing the maximum concentration of **RPF101** used in the steady-state fluorescence studies (1.32 × 10^−5^ M) at room temperature (ca. 298 K). The instrument response function (IRF) was obtained through the suspension of titanium dioxide (TiO_2_) in a mix of glycerol and distilled water (proportion 1:5).

### 2.4. Synchronous Fluorescence Measurements

Synchronous fluorescence (SF) spectra were performed in a model Xe900 Edinburgh Instruments fluorimeter (Edinburgh, UK). Synchronous fluorescence spectrum for HSA (1.00 × 10^−5^ M) was recorded with increasing concentration of **RPF101** in the same concentration range that was used in the steady-state fluorescence studies. The spectra were recorded in the 245–320 nm range by setting constant wavelength interval, Δλ = 60 nm and Δλ = 15 nm for tryptophan and tyrosine residues, respectively, at room temperature (ca. 298 K).

### 2.5. Zeta Potential Measurements

The surface charge of HSA in the absence and presence of **RPF101** was characterized in terms of zeta potential (ZP), using a NanoBrookZetaPALS (Brookhaven Instruments, New York, NY, USA). All measurements were performed with 10 runs at room temperature (ca. 298 K) and the results were reported in terms of ZP ± SD, where SD is the standard deviation. The ZP was measured for HSA solution (1.00 × 10^−5^ M in PBS solution) without and in the presence of the maximum ligand concentration being used in the steady-state fluorescence experiments (1.32 × 10^−5^ M) at room temperature (ca. 298 K).

### 2.6. Circular Dichroism Measurements

Circular dichroism (CD) spectra were measured on a Jasco J-815 spectrometer (Easton, MD, USA), in a 1 cm quartz cell, employing a Jasco PFD-425S15F thermostated cuvette holder. All the spectra were recorded with appropriate background corrections. CD spectra were measured in the 200–260 nm range, at 310 K, using a 1.0 cm path length quartz cuvette, with a 1.0 nm step resolution, and a response time of 1.0 s. The spectra were collected and averaged over three scans. All spectra were baseline corrected by a control sample (3.0 mL of buffer + 40 µL of methanol). Firstly, the spectrum of a free HSA solution (1.00 × 10^−6^ M in PBS solution) was recorded and then the spectrum resulting from the addition of the amount of **RPF101** to obtain the maximum concentration used in the steady-state fluorescence experiments (1.32 × 10^−5^ M) to the HSA solution was also recorded.

### 2.7. Drug Displacement Experiment

Competitive binding studies were carried out with three probes widely employed for the characterization of binding sites in HSA, i.e., warfarin, ibuprofen, and digitoxin for site I, II, and III, respectively [18]. HSA and site probes were used at a fixed concentration (1.00 × 10^−5^ M—proportion 1:1) and the fluorescence quenching titration with **RPF101** was performed, as described previously in the steady-state fluorescence quenching method at 310 K.

### 2.8. Molecular Docking Studies

The crystallographic structure of HSA was obtained from the Protein Data Bank (PDB) with access code 1N5U [19]. This structure has a resolution of 1.90 Å. The **RPF101** structure was built and energy-minimized with the density functional theory (DFT), method Becke-3-Lee Yang Parr (B3LYP) with the standard 6-31G* basis set available in the Spartan’14 program (Wavefunction, Inc., Irvine, CA, USA). Molecular docking was performed with the GOLD 5.2 program (CCDC) [20]. The scoring function used was ‘*ChemPLP*’, which is the default function of the GOLD 5.2 program [21]. Hydrogen atoms were added to HSA according to the data inferred by GOLD 5.2 program on the ionization and tautomeric states. Docking interaction cavity in the protein was established with a 10 Å radius from the Trp-214 residue. The number of genetic operations (crossover, migration, mutation) in each docking run that was used in the search procedure was set to 100,000. The figure of the best docking pose for each sample was generated by the PyMOL Delano Scientific LLC program. Further details can be found in previous publications [17,22].

## 3. Results

### 3.1. Steady-State and Time-Resolved Fluorescence Quenching

**Figure 2:**
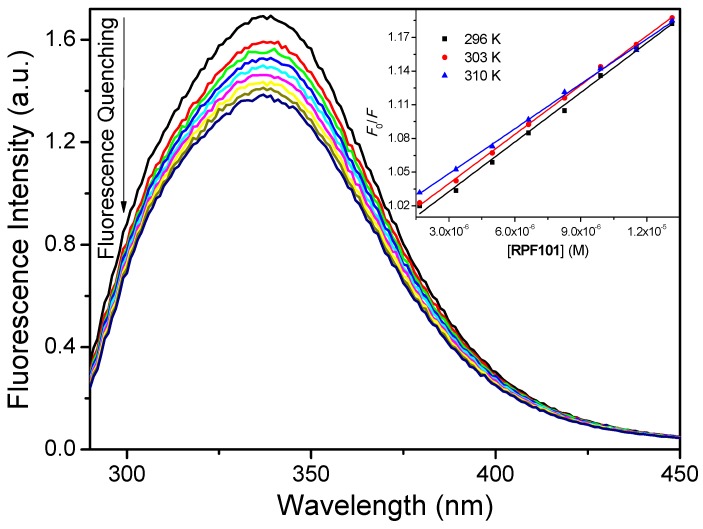
Steady-state fluorescence emission spectra for free human serum album (HSA) and its fluorescence quenching by addition of successive aliquots of **RPF101** in phosphate-buffered saline (PBS) solution at 310 K. [HSA] = 1.00 × 10^−5^ M and [**RPF101**] = 0.17; 0.33; 0.50; 0.66; 0.83; 0.99; 1.15; 1.32 × 10^−5^ M. *Inset*: Stern-Volmer plots for the fluorescence quenching of HSA by **RPF101** at 296, 303 and 310 K.

According to the literature, of the twenty naturally occurring amino acids that make all proteins, three of them contain aromatic ring side chains, and therefore are intrinsically able to display fluorescence emission: Trp, Tyr, and phenylalanine (Phe). Upon 280 nm excitation, the fluorescence from albumin is originated mainly from the dominant source of absorption and emission—the indole group of tryptophan residues (ε = 5600 M^−1^ cm^−1^)—compared with phenylalanine (ε = 200 M^−1^ cm^−1^) or tyrosine (ε = 1400 M^−1^ cm^−1^) [23]. Human serum albumin has a single tryptophan residue (Trp-214) that is located in a hydrophobic cavity inside the subdomain IIA, known as Sudlow’s site I. The intrinsic fluorescence emitted by Trp-214 is very sensitive to the environment around this amino acid residue. Tryptophan fluorescence has been employed frequently in the study of HSA interaction with different biological molecules, i.e., fatty acids and commercial and potential drugs [12,19]. Previous studies have shown that a solution of HSA in PBS (pH = 7.4) has a strong fluorescence emission at 340 nm when excited at 280 nm [24]. Successive addition of **RPF101** to the HSA solution led to the effective fluorescence quenching of Trp-214, while both emission maximum and peak shape remained largely unchanged (Figure 2). This result indicates that the ligand can quench the internal fluorescence of the albumin and the absence of significant blue or red shift in the maximum of fluorescence is indicative that **RPF101** does not change the environment in the vicinity of the fluorophores [21].

A variety of molecular interactions can result in two different quenching mechanisms of a fluorescent species, i.e., dynamic or static. These interactions include ground-state complex formation, collisional quenching, excited state reactions, molecular rearrangement and energy transfer. Dynamic and static quenching can be distinguished by their different dependence on temperature and viscosity, or preferably by lifetime measurements [25]. In general, Stern-Volmer analysis (Equation (2) and *inset* in the Figure 2), as well as the known relationship between *k_q_* and *K_SV_* (Equation (3)), is useful in the estimation of the accessibility of the quencher molecule to the tryptophan residue in proteins as well as in the understanding of the mechanism that is involved in the quenching process [26]:

(2) $$\frac{F_{0}}{F}=1+k_{q}\tau_{0}\left[Q\right]=1+K_{SV}\left[Q\right]$$

(3) $$k_{q}=\frac{K_{SV}}{\tau_{0}}$$

where, *F*_0_ and *F* are the fluorescence intensities of HSA without and in the presence of **RPF101**, respectively. *K_SV_* and *k_q_* are the Stern-Volmer quenching constant and bimolecular quenching rate constant, respectively. [*Q*] is the **RPF101** concentration and *τ_0_* is the fluorescence lifetime of HSA without the presence of **RPF101**—the measured mean value for the fluorescence lifetime of HSA was (5.78 ± 0.15) × 10^−9^ s; see time-resolved fluorescence studies.

**Figure 3:**
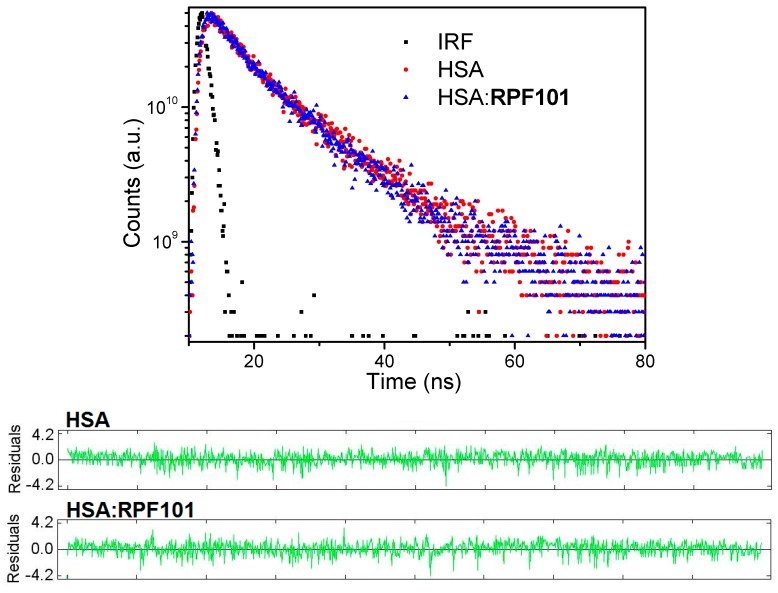
Time-resolved fluorescence decays and residuals for HSA without and in the presence of **RPF101** in PBS solution, at room temperature. [HSA] = 1.00 × 10^−5^ M and [**RPF101**] = 1.32 × 10^−5^ M.

Table 1 shows the *K_SV_* and *k_q_* values for HSA:**RPF101**. Since the obtained *K_SV_* values decrease with the increase of temperature and the bimolecular quenching rate constant values (*k_q_* ≈ 10^12^ M^−1^ s^−1^) are three orders of magnitude larger than the diffusion rate constant (*k_diff_* ≈ 7.40 × 10^9^ M^−1^ s^−1^, at 298 K, according to Smoluchowski-Stokes-Einstein theory) [27], the probable mechanism of fluorescence quenching is static, implying a ground-state association between the fluorophore (albumin) and the quencher (**RPF101**) [28]. In order to further confirm which type of fluorescence quenching mechanism is involved on the HSA:**RPF101** interaction, time-resolved fluorescence measurements were performed for HSA without and in the presence of **RPF101**. Figure 3 depicts the fluorescence lifetime decay profiles for the native HSA and HSA associated with **RPF101**.

The fluorescence decays of HSA upon 280 nm excitation, at pH 7.4, were well-fitted assuming two exponentials having lifetimes of 1.52 ± 0.11 ns (22.0%) and 5.78 ± 0.15 ns (78.0%) (χ^2^ = 1.102). The experimental fluorescence lifetimes are in good agreement with the literature [29,30,31]. No significant changes in the HSA fluorescence lifetimes were observed in the presence of **RPF101**, when values of 1.58 ± 0.14 ns and 5.72 ± 0.13 ns (χ^2^ = 1.135) were obtained. Thus, the ratio between the fluorescence lifetimes of HSA in the absence and presence of **RPF101** are close to unity, suggesting that the fluorescence quenching occurs through a static mechanism [32]. These results are in agreement with those that were obtained above by the Stern-Volmer analysis (Table 1).

There are two basic mechanisms for electronic energy transfer: electron exchange (Dexter) and dipole interaction (Förster). The efficiency of the former falls off exponentially with the distance between fluorophore and quencher, so it only operates at very short distances, essentially by contact; for the Förster mechanism, efficiency decreases with 1/r^6^, so it is still operational at ~10 Å [33]. Thus, Förster resonance energy transfer (FRET) is a non-radiative process: through-space coupling between the oscillating electronic dipole of the excited energy donor (Trp fluorophore in HSA) and that of the ground-state acceptor (quencher-**RPF101**) results in the de-excitation of the former and electronic excitation of the latter: transference of a “virtual”, rather than “real” photon. As a result, the donor fluorophore returns to its ground state, without emission of fluorescence, while the acceptor quencher is promoted to its excited state [21]. Therefore, the quenching of the fluorophore by FRET can occurs if there is an overlap between the fluorophore emission and quencher absorption spectra. In Figure S3 in the Supplementary Material, such overlap between the fluorescence emission spectrum of HSA and the absorption spectrum of **RPF101** can be clearly seen, indicating the possible occurrence of an energy transfer process between the fluorophore in protein and the ground state of **RPF101 [34]**. But, the absence of any change in the fluorescence lifetime of HSA after the addition of **RPF101** indicates that FRET is not operating in the present case [23].

**Figure 4:**
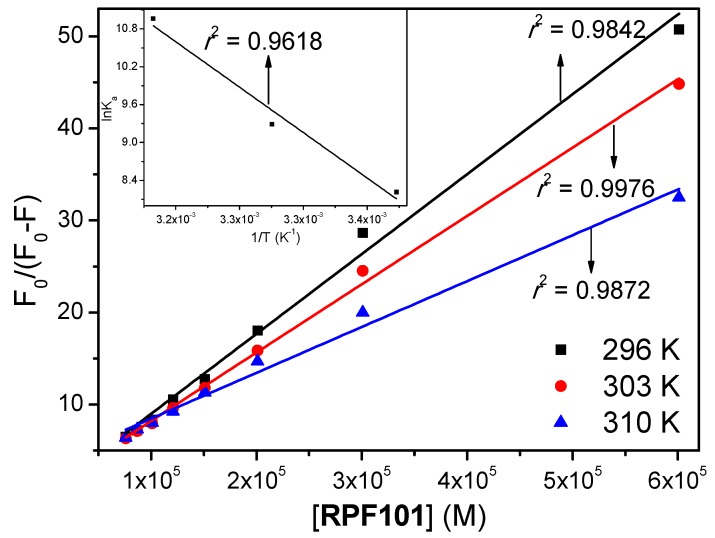
Modified Stern-Volmer plots for the association HSA:**RPF101** in PBS solution at 296, 303 and 310 K. *Inset*: Van’t Hoff plot for HSA:**RPF101** at three different temperatures. [HSA] = 1.00 × 10^−5^ M and [**RPF101**] = 0.17; 0.33; 0.50; 0.66; 0.83; 0.99; 1.15; 1.32 × 10^−5^ M.

From the pharmacological point of view, if the drugs are metabolized and excreted from the body too fast because of low protein binding, they will not be able to provide their therapeutic effects. On the other hand, if drugs bind too strongly to protein and are metabolized and excreted too slowly, the in vivo half-life of these drugs can increase excessively, and this may lead to undesired side effects and/or toxicity [35]. To obtain information about the association between HSA and **RPF101**, the modified Stern-Volmer binding constant (*K_a_*) was calculated while employing the Figure 4 and Equation (4):

(4) $$\frac{F_{0}}{F_{0}-F}=\frac{1}{fK_{a}[Q]}+\frac{1}{f}$$

where, *F*_0_ and *F* are the fluorescence intensities of HSA without and in the presence of **RPF101** at 340 nm, respectively. [*Q*] is the **RPF101** concentration and *f* is the fraction of the initial fluorescence that is accessible to quenchers (*f* ≈ 1.00). The *K_a_* values for the association HSA:**RPF101** are in the range of 10^3^–10^4^ M^−1^, showing a moderate interaction between the potential drug **RPF101** and HSA [36,37] (Table 1), suggesting that **RPF101** can be stored and carried by the protein in the human bloodstream [38,39]. The increase of *K_a_* values with the increase of temperature indicates that the protein structure can better accommodate the ligand at 310 K (human body temperature) than at 296 K, which is probably due to binding pocket being more accessible by the quenchers at 310 K.

In general, the main interaction forces between endogenous and exogenous molecules with proteins can include hydrophobic interaction, hydrogen bond, van der Waals, and electrostatic forces. These intermolecular interactions can be related to the thermodynamic parameters Δ*G*°, Δ*H*° and Δ*S*° [40], which can be obtained applying the van’t Hoff analysis (Equation (5) and *inset* in the Figure 4) and the Gibbs free energy analysis (Equation (6)) [39].

(5) $$\ln K_{a}=-\frac{\Delta H^{{}^{\circ}}\;}{RT}+\frac{\Delta S^{{}^{\circ}}}{R}$$

(6) $$\Delta G^{{}^{\circ}}=\Delta H^{{}^{\circ}}-T\Delta S^{{}^{\circ}}$$

where, Δ*H*°, Δ*S*°, Δ*G*° are the enthalpy, entropy, and Gibbs free energy change, respectively. *R* is the gas constant (*R* = 8.314 × 10^−3^ kJ/mol K), *T* is the temperature (296, 303 and 310 K), and *K_a_* the modified Stern-Volmer binding constant. Negative values for Δ*G*° are in further accord with the spontaneity of the binding process between HSA and **RPF101**. The unfavorable positive Δ*H*° can be compensated by the positive Δ*S*°, which indicate a hydrophobic interaction [41] and suggest that the binding process is entropically driven [42].

### 3.2. Synchronous Fluorescence Spectroscopy

**Figure 5:**
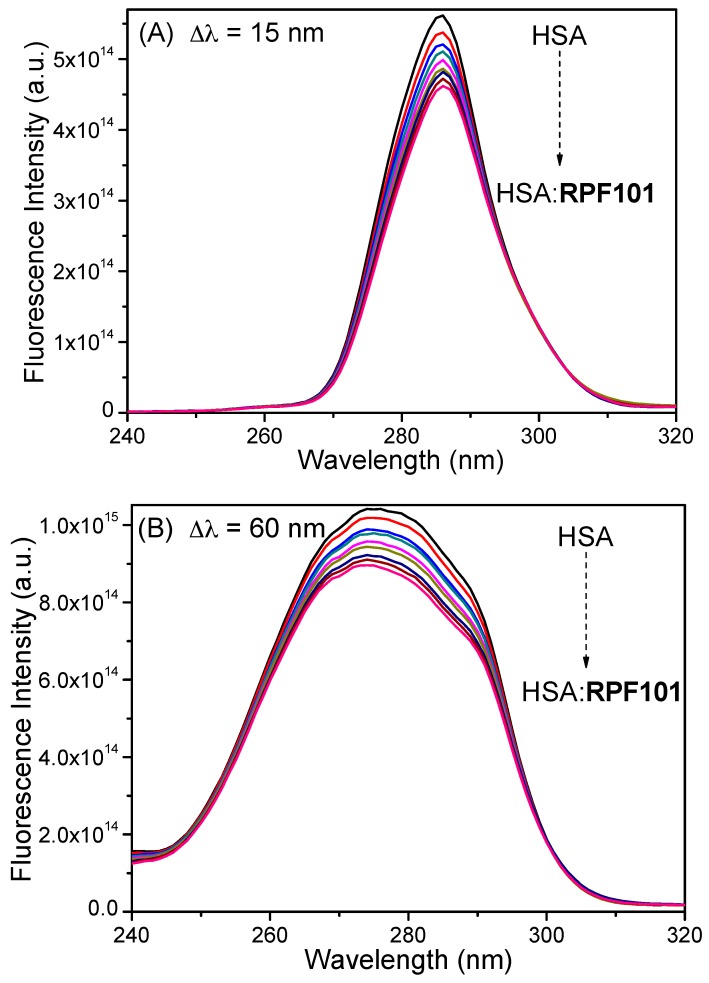
Synchronous fluorescence spectra for HSA without and in the presence of **RPF101** at Δλ = 15 nm (**A**) and Δλ = 60 nm (**B**) in PBS solution. [HSA] = 1.00 × 10^−5^ M and [**RPF101**] = 0.17; 0.33; 0.50; 0.66; 0.83; 0.99; 1.15; 1.32 × 10^−5^ M.

In the synchronous fluorescence (SF) spectra, the sensitivity that is associated with fluorescence is maintained, while several advantages are available: spectral simplification, spectral bandwidth reduction, and avoidance of different perturbing effects [18]. By scanning the excitation and emission monochromators simultaneously, while maintaining a constant wavelength interval (Δλ) between them, characteristic information about the molecular environment in the vicinity of a chromophore can be obtained [43]. The SF spectra of HSA for tyrosine (Δλ = 15 nm) and tryptophan residues (Δλ = 60 nm) in the presence of various concentrations of **RPF101** are shown in Figure 5. This figure clearly shows that in both cases there is no significant Stokes’ shift at the maximum fluorescence emission after successive additions of **RPF101** to the HSA solution, suggesting that there is no significant change on the HSA structure upon ligand binding that can perturb the microenvironment around the Tyr and Trp residues [21,44].

### 3.3. Zeta Potential Studies

Changes in the zeta potential (ZP, ζ) for a protein can mainly imply in conformational changes and/or unfolding/denaturation processes on the protein structure. Therefore, the ZP of a protein can be used as an indicator of the protein stability upon ligand binding [45]. The experimental ZP for free HSA was negative (ζ ≈ −7.50 ± 2.76 mV, conductance ≈ 30,232 µS, and electric field ≈ 13.60 V/cm) at pH = 7.4 in PBS buffer solution. On the other hand, upon the addition of **RPF101** (1.32 × 10^−5^ M), the ZP was ζ ≈ −9.50 ± 1.90 mV, conductance ≈ 28,865 µS, and electric field ≈ 14.30 V/cm. It is worth to note that the ZP value for HSA before and after addition of **RPF101** is the same inside the experimental error of the measurements, indicating that there is no significant structural change on the protein surface upon ligand addition [46].

### 3.4. Change on the Protein Secondary Structure Induced by **RPF101** Binding

**Figure 6:**
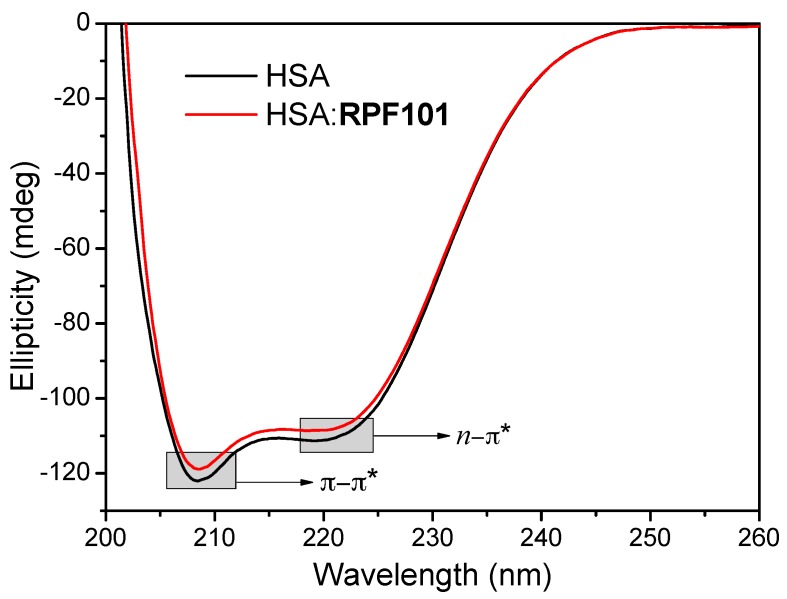
Circular dichroism spectra for HSA without and in the presence of **RPF101** in PBS buffer solution (pH = 7.4) at 310 K. [HSA] = 1.00 × 10^−6^ M and [**RPF101**] = 1.32 × 10^−5^ M.

Circular dichroism (CD) spectra of HSA exhibited negative bands at 208 and 222 nm, corresponding to π-π* and *n*-π* transitions, respectively, which are characteristic of the *α-helix* structure units of the protein [47]. Upon the addition of **RPF101** (1.32 × 10^−5^ M) to the albumin solution, a small decrease in the intensity of the absorptions at 208 and 222 nm was observed (Figure 6), indicating a very weak change on the secondary structure of HSA [48].

Circular dichroism results can be expressed in terms of significant molar residual ellipticity (MRE) in degcm^2^/dmol, calculated according to Equation (7):

(7) $$MRE=\frac{\theta }{\left(10.n.l.C_{P}\right)}$$

where, *θ* is the observed ellipticity (mdeg); *n* is the number of amino acid residues (585 to HSA) [49]; *l* is the length of the optical cuvette (1 cm); and *C_p_* is the molar concentration of HSA (1.00 × 10^−6^ M). The loss of helical structure due to ligand binding can also be quantitatively calculated as contents of free and combined HSA from MRE values at 208 and 222 nm, while applying Equations (8) and (9), respectively:

(8) $$\alpha -helix\%=\left[\frac{\left(-MRE_{208}-4000\right)}{\left(33000-4000\right)}\right]\times 100$$

(9) $$\alpha -helix\%=\left[\frac{\left(-MRE_{222}-2340\right)}{30300}\right]\times 100$$

where, *MRE*_208_ and *MRE*_222_ are the significant molar residual ellipticities (degcm^2^/dmol) at 208 and 222 nm, respectively. The *α*-*helix* content of the secondary structure of HSA in the absence of **RPF101** has its maximum at about 57.5% and 53.9% at 208 and 222 nm, respectively, while in the presence of **RPF101**, the *α*-*helix* content decreased at about 55.3% and 52.1% at 208 and 222 nm, respectively. Thus, it may be concluded from the CD results that the binding HSA:**RPF101** can occur with a very weak change on the secondary structure of the protein [36,50].

### 3.5. Competitive Binding Studies

In general, the main regions of small molecules binding sites on HSA are located in the hydrophobic cavities in subdomains IIA and IIIA, which are also referred as Sudlow’s site I and site II, respectively, according to the terminology that was proposed by Sudlow and coworkers [51]. Furthermore, the hydrophilic cavity located in subdomain IB, which can be referred as site III, is also considered as a possible protein pocket for small molecules [18]. In order to identify the main protein cavity for the association HSA:**RPF101**, competitive binding studies were performed at 310 K using different site probes, like warfarin, ibuprofen, and digitoxin for sites I, II, and III, respectively [36].

**Figure 7:**
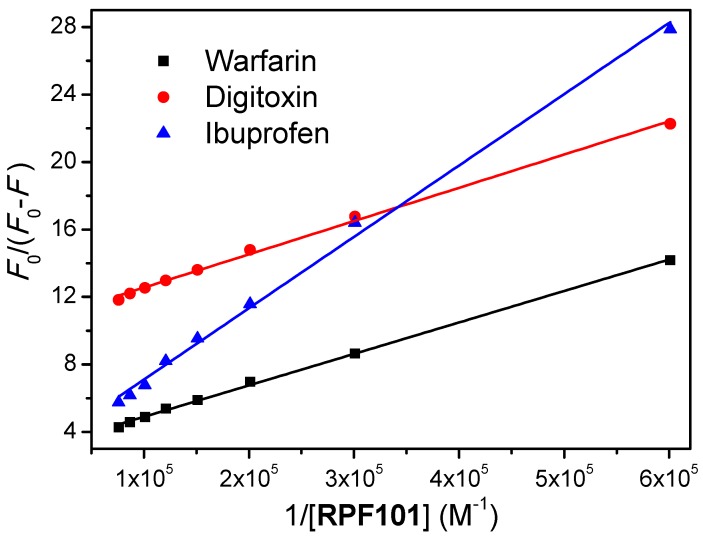
Modified Stern-Volmer plot for the interaction HSA:**RPF101** in the presence of each site marker: warfarin, ibuprofen and digitoxin (1.00 × 10^−5^ M). [HSA] = [warfarin] = [ibuprofen] = [digitoxin] = 1.00 × 10^−6^ M and [**RPF101**] = 0.17; 0.33; 0.50; 0.66; 0.83; 0.99; 1.15; 1.32 × 10^−5^ M.

The *K_a_* values determined by Equation (4) and Figure 7 for HSA:**RPF101** in the presence of 1.00 × 10^−5^ M warfarin, ibuprofen, or digitoxin at 310 K are shown in Table 2. From the results that are shown in this table, it can be seen that the HSA:**RPF101** binding in the presence of warfarin was reduced by 71.8% when compared to HSA:**RPF101,** without any site marker. On the other hand, in the presence of digitoxin and ibuprofen, the decrease in *K_a_* value was much smaller−21.5% and 11.9%, respectively. This is a clear indication that **RPF101** competes with warfarin for the subdomain IIA, where the Trp-214 residue can be found [18,21,36].

### 3.6. Molecular Docking Studies for the Interaction HSA:**RPF101**

The spectroscopic results that are described above indicated that the main binding site for **RPF101** in the HSA structure is Sudlow’s site I, in subdomain IIA, where Trp-214 residue can be found. Thus, this protein pocket was chosen for performing computational experiments that aimed to provide a more detailed (atomistic view) of the binding interaction.

**Figure 8:**
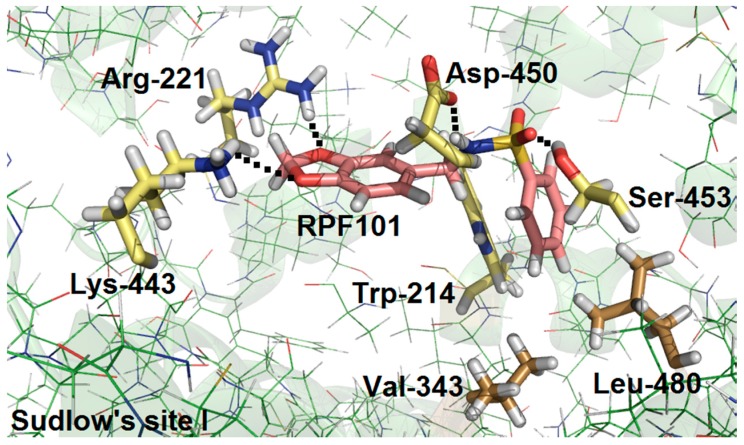
The best score pose for the interaction HSA:**RPF101** in Sudlow’s site I, as obtained by molecular docking (*ChemPLP* function). **RPF101** structure, selected hydrophilic and hydrophobic amino acid residues are represented in beige, yellow and brown, respectively. Green color is the HSA structure (PDB:1N5U). Elements colors: hydrogen, oxygen, nitrogen, and sulfur are represented in white, red, blue, and goldenrod, respectively. Arginine: Arg; aspartic acid: Asp; lysine: Lys; tryptophan: Trp; serine: Ser; leucine: Leu; valine: Val.

The molecular docking results suggested that hydrogen bonding and hydrophobic interactions are the main forces for the association HSA:**RPF101** (Figure 8). Both oxygen atoms of the methylenedioxolyl moiety of **RPF101** are acceptors of hydrogen bonding for arginine-221 (Arg-221) and lysine-443 (Lys-443) residues, within a distance of 2.08 Å and 3.65 Å, respectively. The hydrogen atom of the sulfonamide linker of **RPF101** is a possible donor for hydrogen bonding with the aspartic acid-450 (Asp-450) residue within a distance of 2.10 Å, whereas the sulfone can interact with the hydroxyl group of the serine-453 (Ser-453) side chain via hydrogen bonding within a distance of 1.82 Å.

Finally, molecular docking results also suggested hydrophobic interactions via π-stacking between the Trp-214 residue and the phenyl ring of **RPF101** attached to the sulfonamide linker, within a distance of 3.50 Å. This very same aromatic ring can interact with the amino acid residues valine-343 (Val-343) and leucine-480 (Leu-480) within distances of 2.78 Å and 1.60 Å, respectively. Overall, the molecular docking results are in good agreement with the experimental data discussed above.

## 4. Discussion

In the literature, there is a reasonable amount or work describing the interaction between HSA:natural products and HSA:natural products analogues, such as mangiferin [52], kaempferol [49], wogonin [39], pheophytin [26], and flanovoids [21,53]. In the case of natural products that are extracted from peppers and its analogues, it was only explored the interaction between serum albumin and 1-piperoyl piperidine (piperine) [54,55]. Since there are few investigations on the interaction between serum albumin and natural products analogues from peppers components, as well as the application of multi-spectroscopic techniques combined with theoretical methods can be used as an initial process for the evaluation of some pharmacokinetic profile of potential drugs, the present discussion is focused on the interaction HSA:**RFP101** by circular dichroism, steady-state, time-resolved, and synchronous fluorescence combined with zeta potential and theoretical calculations.

The Stern-Volmer analysis applied in the steady-state fluorescence data indicated a decrease in the *K_SV_* values with increasing of temperature and the *k_q_* values are three orders of magnitude higher than *k_diff_*. These observations can indicate a ground state association between HSA and **RPF101** (static fluorescence quenching mechanism). To get more insight into the fluorescence mechanism of **RPF101** to serum albumin, the time-resolved fluorescence technique was employed. This technique revealed that the fluorescence decays for HSA are composed by two different lifetimes being the second one the most contribution. Since the fluorescence lifetimes for HSA without and in the presence of the ligand (**RPF101**) are the same inside the experimental error, i.e., τ_2_ = 5.78 ± 0.15 and τ_2_ = 5.72 ± 0.13 ns, respectively, the static fluorescence quenching mechanism can be confirmed.

The binding affinity of any substance to serum albumin is one of the major factors that determine the pharmacokinetics i.e., time course of drug absorption, distribution, metabolism, and excretion. Since the modified Stern-Volmer binding constant values are in the order of 10^3^–10^4^ M^−1^, there is an indicative of moderate binding affinity between HSA and **RPF101** [56]. Since, Δ*G*° values for the interaction HSA:**RPF101** are negative, thus indicating a spontaneous process. The positive values for Δ*H*° and Δ*S*° are indicative of a binding process that is controlled by entropy [31]. According to the Gibbs free energy equation, a positive enthalpy change is not favorable for the spontaneity of the binding process, unlike a positive entropy change that leads to a more negative value for the Gibbs free energy. From Ross and Subramanian theory [41] positive values for enthalpy and entropy change suggest hydrophobic interaction as the main intermolecular force that is involved in the binding process. These association parameters indicated that **RPF101** presented similar binding ability toward HSA when compared to piperine [55].

The steady-state fluorescence spectrum of HSA presented maximum fluorescence emission at 340 nm. Upon successive additions of **RPF101** to HSA solution fluorescence quenching can be observed without any blue or red shift (Stokes’ shift), indicating that the ligand binding does not perturb significantly the fluorophore environment [21,22]. In order to confirm this result, synchronous fluorescence (SF) spectra was carried out at Δλ = 15 and 60 nm. In the SF spectra, a decrease in fluorescence intensity without any shift is an indicative that there is no significant changes on the HSA structure upon ligand binding, which can perturb the microenvironment around Tyr and Trp residues [18].

The binding of **RPF101** to HSA structure could cause some modifications on the surface and secondary structure of the albumin, which could reflect in an inactivation of the protein activity. According to zeta potential results (ζ ≈ −7.50 ± 2.76 and −9.50 ± 1.90 mV, for HSA and HSA:**RPF101**, respectively), there is a clear indication that the binding of the ligand does not perturb the protein surface (ζ without and in the presence of **RPF101** are the same inside the experimental error) [46]. Circular dichroism results indicated a very weak perturbation on the secondary structure of the albumin upon ligand binding (variation of 2.20% and 1.80% at 208 and 222 nm, respectively) [37]. Thus, besides the presence of the capsaicin analogue does not perturb the microenvironment around Trp and Tyr residues it does not significantly perturb the surface and secondary structure of the albumin.

HSA structure has three main binding sites, which are located in the subdomain IIA (site I), IIIA (site II), and IB (site III) [51,56]. Competitive binding studies in the presence of three commercial site markers (warfarin, ibuprofen, and digitoxin) suggested site I, which is also known as Sudlow’s site I, as the main protein binding pocket for **RPF101** (*K_a_* value without and in the presence of warfarin changed 71.8% at 310 K). The same binding pocket was also identified for piperine, which has the same (1,3)-benzodioxolyl moiety and close ClogP values (theoretical octanol/water partition coefficient) as compared to **RPF101** (2.15 and 2.78, respectively) [4,55]. In order to offer an atomic view of the interaction HSA:**RPF101**, theoretical calculations via molecular docking were carried out. Molecular docking results suggested hydrophobic interactions and hydrogen bonding as the main binding forces in the association HSA:**RPF101** in the subdomain IIA. There are chemical groups in the ligand structure that are possible acceptors for hydrogen bonding with Arg-221, Lys-443, and Ser-453 residues. On the other hand, the amino acid residue Asp-450 is a potential acceptor for hydrogen bonding with the ligand structure. Finally, hydrophobic interactions were also suggested between **RPF101** and three amino acid residues: Trp-214, Val-343, and Leu-480. Note that the experimental data obtained from steady-state fluorescence measurements indicated the interaction HSA:**RPF101** as essentially entropically driven, while molecular docking results, which give evidences from an atomic point of view, suggested entropic and enthalpic contributions. Thus, **RPF101** can interact with albumin essentially controlled by entropic effects, however, the enthalpic contribution can also be involved in this association. Overall, the capsaicin analogue presented good binding ability toward HSA, indicating high probability to be carried in the human bloodstream.

## 5. Conclusions

Fluorescence quenching studies of HSA by **RPF101** showed *K_SV_* and *k_q_* values that led us to conclude that the fluorescence quenching occurs via a static mechanism, which indicates the presence of a ground state association HSA:**RPF101**. This conclusion was supported by time-resolved fluorescence results. The interaction HSA:**RPF101** is moderate (*K_a_* ≈ 10^3^–10^4^ M^−1^), entropically driven, spontaneous and does not significantly change the potential surface of the protein, as well as the environment around tyrosine and tryptophan residues. The CD results indicated that upon ligand binding there is a very weak perturbation on the secondary structure of the albumin. Competitive binding studies indicated Sudlow’s site I—located in the subdomain IIA—as the main protein pocket for this association. Molecular docking results suggested that the ligand interacts via hydrogen bonding with Arg-221, Lys-443, Asp-450, and Ser-453 residues and also via hydrophobic interactions with Trp-214, Val-343, and Leu-480 residues. At comparing spectroscopic and molecular docking results the interaction HSA:**RPF101** is essentially controlled by entropic effects, however, the enthalpic contribution can also be an evidences involved in this association. Overall, the potential drug **RPF101** can be carried and distributed by HSA in the human bloodstream.

## Figures and Tables

**Table 1:** Stern-Volmer quenching constant (*K_SV_*), bimolecular quenching rate constant (*k_q_*), modified Stern-Volmer binding constant (*K_a_*) and thermodynamic parameters (Δ*H*°, Δ*S*°, and Δ*G*°) for HSA:**RPF101** at 296, 303, and 310 K ^a^.

T (K)	*K_SV_* (M^−1^)	*k_q_* (M^−1^ s^−1^)	*K_a_* (M^−1^)	Δ*H*° (kJ/mol)	Δ*S*° (kJ/molK)	Δ*G*° (kJ/mol)
296	(1.47 ± 0.04) × 10^4^	2.54 × 10^12^	(3.70 ± 0.26) × 10^3^			−20.6
303	(1.45 ± 0.02) × 10^4^	2.50 × 10^12^	(1.08 ± 0.26) × 10^4^	149 ± 20	0.573 ± 0.069	−24.6
310	(1.33 ± 0.02) × 10^4^	2.30 × 10^12^	(5.79 ± 0.26) × 10^4^			−28.6

a: *r*^2^ for *K_SV_* and *k_q_*: 0.9988–0.9945; *r*^2^ for *K_a_*: 0.9976–0.9842; *r*^2^ for Δ*H*°, Δ*S*° and Δ*G*°: 0.9618.

**Table 2:** *K_a_* value for the interaction HSA:**RPF101** without and in the presence of warfarin, ibuprofen, or digitoxin at 310 K ^a^.

*Sample*	K_a_ (M^−1^)	K_a_ (M^−1^)	K_a_ (M^−1^)	K_a_ (M^−1^)
	Without Site Marker	Presence Warfarin	Presence Ibuprofen	Presence Digitoxin
**RPF101**	(5.79 ± 0.26) × 10^4^	(1.63 ± 0.26) × 10^4^	(5.10 ± 0.26) × 10^4^	(4.55 ± 0.26) × 10^4^

a: r^2^ for *K_a_*: 0.9960–0.9987.

## Supplementary Materials

The following are available online at http://www.mdpi.com/2218-273X/8/3/78/s1, Figure S1: UV-Vis spectra for HSA and **RPF101** in PBS solution. [HSA] = 1.00 × 10^−5^ M and [**RPF101**] = 1.32 × 10^−5^ M. Figure S2: (A) Steady-state fluorescence emission spectra for HSA without and in the presence of 40 µL of methanol at 310 K. (B) CD spectra for HSA without and in the presence of 40 µL of methanol at 310 K. Figure S3: Overlap between UV-Vis spectrum of **RPF101** and steady-state fluorescence emission spectrum of HSA at 310 K. [HSA] = [**RPF101**] = 1.00 × 10^−5^ M.

## References

[1] Clark R., Lee S. (2016). Anticancer properties of capsaicin against human cancer. Anticancer Res..

[2] Lee J.H., Lee Y., Ryu H.C., Kang D.W., Lee J., Lazar J., Pearce L.V., Pavlyukovets V.A., Blumberg P.M., Choi S. (2011). Structural insights into transient receptor potential vanilloid type 1 (TRPV1) from homology modeling, flexible docking, and mutational studies. J. Comput. Aided Mol. Des..

[3] Yong Y.L., Tan L.T.H., Ming L.C., Chan K.G., Lee L.H., Goh B.H., Khan T.M. (2017). The effectiveness and safety of topical capsaicin in postherpetic neuralgia: A systematic review and meta-analysis. Front. Pharmacol..

[4] Sá-Júnior P.L., Pasqualoto K.F.M., Ferreira A.K., Tavares M.T., Damião M.C.F.C.B., De Azevedo R.A., Câmara D.A.D., Pereira A., Souza D.M., Parise-Filho R. (2013). RPF101, a new capsaicin-like analogue, disrupts the microtubule network accompanied by arrest in the G2/M phase, inducing apoptosis and mitotic catastrophe in the MCF-7 breast cancer cells. Toxicol. Appl. Pharmacol..

[5] Damião M.C., Pasqualoto K.F., Ferreira A.K., Teixeira S.F., Azevedo R.A., Barbuto J.A., Berl F.P., Franchi-Junior G.C., Nowill A.E., Tavares M.T. (2014). Novel capsaicin analogues as potential anticancer agents: Synthesis, biological evaluation, and *In silico* approach. Archiv der Pharmazie.

[6] Tavares M.T., Pasqualoto K.F.M., Van de Streek J., Ferreira A.K., Azevedo R.A., Damião M.C.F.C.B., Rodrigues C.P., de-Sá-Júnior P.L., Barbuto J.A.M., Parise-Filho R. (2015). Synthesis, characterization, *in silico* approach and *in vitro* antiproliferative activity of RPF151, a benzodioxole sulfonamide analogue designed from capsaicin scaffold. J. Mol. Struct..

[7] Ferreira A.K., Tavares M.T., Pasqualoto K.F.M., Azevedo R.A., Teixeira S.F., Ferreira-Junior W.A., Bertin A.M., de-Sá-Junior P.L., Barbuto J.A.M., Figueiredo C.R. (2015). RPF151, a novel capsaicin-like analogue: In vitro studies and in vivo preclinical antitumor evaluation in a breast cancer model. Tumor Biol..

[8] Peters T. (1985). Serum albumin. Adv. Protein Chem..

[9] Yeggoni D.P., Gokara M., Manidhar D.M., Rachamallu A., Nakka S., Reddy C.S., Subramanyam R. (2014). Binding and molecular dynamics studies of 7-hydroxycoumarin derivatives with human serum albumin and its pharmacological importance. Mol. Pharm..

[10] Carter D.C., Ho J.X. (1994). Structure of serum albumin. Adv. Protein Chem..

[11] Carter D.C., He X.-M., Munson S.H., Twigg P.D., Gernert K.M., Broom M.B., Miller T.Y. (1989). Three-dimensional structure of human serum albumin. Science.

[12] Shahsavani M.B., Ahmadi S., Aseman M.D., Nabavizadeh S.M., Alavianmehr M.M., Yousef R. (2016). Comparative study on the interaction of two binuclear Pt (II) complexes with human serum albumin: Spectroscopic and docking simulation assessments. J. Photochem. Photobiol. B Biol..

[13] Kratz F. (2008). Albumin as a drug carrier: Design of prodrugs, drug conjugates and nanoparticles. J. Control. Release.

[14] Danhier F., Feron O., Préat V. (2010). To exploit the tumor microenvironment: Passive and active tumor targeting nanocarriers for anti-cancer drug delivery. J. Control. Release.

[15] Desai N., Trieu V., Yao Z., Louie L., Ci S., Yang A., Tao C., De T., Beals B., Dykes D. (2006). Increased antitumor activity, intratumor paclitaxel concentrations, and endothelial cell transport of cremophor-free, albumin-bound paclitaxel, ABI-007, compared with cremophor-based paclitaxel. Clin. Cancer Res..

[16] Shao X., Ai N., Xu D., Fan X. (2016). Exploring the interaction between *Salvia miltiorrhiza* and human serum albumin: Insights from herb—drug interaction reports, computational analysis and experimental studies. Spectrochim. Acta Mol. Biomol. Spectrosc..

[17] Chaves O.A., Jesus C.S.H., Cruz P.F., Sant’Anna C.M.R., Brito R.M.M., Serpa C. (2016). Evaluation by fluorescence, STD-NMR, docking and semi-empirical calculations of the *o*-NBA photo-acid interaction with BSA. Spectrochim. Acta Mol. Biomol. Spectrosc..

[18] Sun Z., Xu H., Cao Y., Wang F., Mi W. (2016). Elucidating the interaction of propofol and serum albumin by spectroscopic and docking methods. J. Mol. Liq..

[19] Wardell M., Wang Z., Ho J.X., Robert J., Ruker F., Ruble J., Carter D.C. (2002). The atomic structure of human methemalbumin at 1.9 Å. Biochem. Biophys. Res. Commun..

[20] The Cambridge Crystallographic Data Centre (CCDC). http://www.ccdc.cam.ac.uk/solutions/csd-discovery/components/gold/.

[21] Chaves O.A., Cesarin-Sobrinho D., Sant’Anna C.M.R., de Carvalho M.G., Suzart L.R., Catunda-Junior F.E.A., Netto-Ferreira J.C., Ferreira A.B.B. (2017). Probing the interaction between 7-*O*-β-d-glucopyranosyl-6-(3-methylbut-2-enyl)-5,4’-dihydroxyflavonol with bovine serum albumin (BSA). J. Photochem. Photobiol. A Chem..

[22] Chaves O.A., Santos M.R.L., Oliveira M.C.C., Sant’Anna C.M.R., Ferreira R.C., Echevarria A., Netto-Ferreira J.C. (2018). Synthesis, tyrosinase inhibition and transportation behavior of novel β-enamino thiosemicarbazide derivatives by human serum albumin. J. Mol. Liq..

[23] Lakowicz J.R. (2006). Principles of Fluorescence Spectroscopy.

[24] Alam P., Abdelhameed A.S., Rajpoot R.K., Khan R.H. (2016). Interplay of multiple interaction forces: Binding of tyrosine kinase inhibitor nintedanib with human serum albumin. J. Photochem. Photobiol. B Biol..

[25] Liu F., Wang Y., Lv C., Wang L., Ou J., Wang M., Liu S. (2012). Impact of halogen substituents on interactions between 2-phenyl-2,3-dihydroquinazolin-4(1*H*)-one derivatives and human serum albumin. Molecules.

[26] Chaves O.A., Amorim A.P.O., Castro L.H.E., Sant’Anna C.M.R., de Oliveira M.C.C., Cesarin-Sobrinho D., Netto-Ferreira J.C., Ferreira A.B.B. (2015). Fluorescence and docking studies of the interaction between human serum albumin and pheophytin. Molecules.

[27] Montalti M., Credi A., Prodi L., Gandolfi M.T. (2006). Handbook of Photochemistry.

[28] Chaves O.A., Soares B.A., Maciel M.A.M., Sant’Anna C.M.R., Netto-Ferreira J.C., Cesarin-Sobrinho D., Ferreira A.B.B. (2016). A study of the interaction between *trans*-dehydrocrotonin, a bioactive natural 19-*nor*-clerodane, and serum albumin. J. Braz. Chem. Soc..

[29] Molina-Bolívar J.A., Ruiz C.C., Galisteo-González F., Donnell M.M.-O., Parra A. (2016). Simultaneous presence of dynamic and sphere action component in the fluorescence quenching of human serum albumin by diphthaloylmaslinic acid. J. Lumin..

[30] Sun H., Liu Y., Li M., Han S., Yang X., Liu R. (2016). Toxic effects of chrysoidine on human serum albumin: Isothermal titration calorimetry and spectroscopic investigations. Luminescence.

[31] Chaves O.A., Mathew B., Joy M., Lohidakshan K.K., Marathakam A., Netto-Ferreira J.C. (2018). Introduction of fluorinated environment on metformin. Evaluation of its serum-albumin interaction with molecular modeling studies. J. Mol. Liq..

[32] Bi S., Zhao T., Zhou H., Wang Y., Li Z. (2016). Probing the interactions of bromchlorbuterol-HCl and phenylethanolamine A with HSA by multi-spectroscopic and molecular docking technique. J. Chem. Thermodyn..

[33] Turro N.J. (1991). Modern Molecular Photochemistry.

[34] Wang Q., Liu X., Su M., Shi Z., Sun H. (2015). Study on the interaction characteristics of cefamandole with bovine serum albumin by spectroscopic technique. Spectrochim. Acta Mol. Biomol..

[35] Chaves O.A., Jesus C.S.H., Henriques E.S., Brito R.M.M., Serpa C. (2016). In-situ ultra-fast heat deposition does not perturb serum albumin structure. Photochem. Photobiol. Sci..

[36] Rehman M.T., Shamsi H., Khan A.U. (2014). Insight into the binding mechanism of imipenem to human serum albumin by spectroscopic and computational approaches. Mol. Pharm..

[37] Chaves O.A., Mathew B., Cesarin-Sobrinho D., Lakshminarayanan B., Joy M., Mathew G.E., Suresh J., Netto-Ferreira J.C. (2017). Spectroscopic, zeta potential and molecular docking analysis on the interaction between human serum albumin and halogenated thienyl chalcones. J. Mol. Liq..

[38] Zhu Y., Zhang R., Wang Y., Ma J., Li K., Li Z. (2014). Biophysical study on the interaction of an anesthetic, vecuronium bromide with human serum albumin using spectroscopic and calorimetric methods. J. Photochem. Photobiol. B Biol..

[39] Tian J.N., Liu J., Hu Z.D., Chen X.G. (2005). Interaction of wogonin with bovine serum albumin. Bioorg. Med. Chem..

[40] Zhang H.-X., Xiong H.-X., Li L.-W. (2016). Investigation on the protein-binding properties of icotinib by spectroscopic and molecular modeling method. Spectrochim. Acta Mol. Biomol. Spectrosc..

[41] Ross P.D., Subramanian S. (1981). Thermodynamics of protein association reactions: Forces contributing to stability. Biochemistry.

[42] De Barros L.S., Chaves O.A., Schaeffer E., Sant’Anna C.M.R., Ferreira A.B.B., Cesarin-Sobrinho D., da Silva F.A., Netto-Ferreira J.C. (2016). Evaluating the interaction between di-fluorinated chalcones and plasmatic albumin. J. Fluor. Chem..

[43] Vignesh G., Sugumar K., Arunachalam S., Vignesh S., James R.A., Arun R., Premkumar K. (2016). Studies on the synthesis, characterization, human serum albumin binding and biological activity of single chain surfactant–cobalt(III) complexes. Luminescence.

[44] Barakat C., Patra D. (2013). Combining time-resolved fluorescence with synchronous fluorescence spectroscopy to study bovine serum albumin-curcumin complex during unfolding and refolding processes. Luminescence.

[45] Hosainzadeh A., Gharanfoli M., Saberi M.R., Chamani J.K. (2012). Probing the interaction of human serum albumin with bilirubin in the presence of aspirin by multi-spectroscopic, molecular modeling and zeta potential techniques: Insight on binary and ternary systems. J. Biomol. Struct. Dyn..

[46] Zhang S., Chen X., Ding S., Lei Q., Fang W. (2016). Unfolding of human serum albumin by gemini and single-chain surfactants: A comparative study. Colloids Surf. A.

[47] Rastegari B., Karbalaei-Heidari H.R., Yousefi R., Zeinali S., Nabavizadeh M. (2016). Interaction of prodigiosin with HSA and β-Lg: Spectroscopic and molecular docking studies. Bioorg. Med. Chem..

[48] Qian Y., Zhou X., Chen J., Zhang Y. (2012). Binding of bezafibrate to human serum albumin: Insight into the non-covalent interaction of an emerging contaminant with biomacromolecules. Molecules.

[49] Matei I., Hillebrand M. (2010). Interaction of kaempferol with human serum albumin: A fluorescence and circular dichroism study. J. Pharm. Biomed. Anal..

[50] Rabbani G., Baig M.H., Lee E.J., Cho W.K., Ma J.Y., Choi I. (2017). Biophysical study on the interaction between eperisone hydrochloride and human serum albumin using spectroscopic, calorimetric, and molecular docking analysis. Mol. Pharm..

[51] Sudlow G., Birkett D.J., Wade D.N. (1976). Further characterization of specific drug binding sites on human serum albumin. Mol. Pharmacol..

[52] Yue Y., Chen X., Qin J., Yao X. (2009). Characterization of the mangiferin-human serum albumin complex by spectroscopic and molecular modeling approaches. J. Pharm. Biomed. Anal..

[53] Wang B., Qin Q., Chang M., Li S., Shi X., Xu G. (2018). Molecular interaction study of flavonoids with human serum albumin using native mass spectrometry and molecular modeling. Anal. Bioanal. Chem..

[54] Yeggoni D.P., Rachamallu A., Kallubai M., Subramanyam R. (2015). Cytotoxicity and comparative binding mechanism of piperine with human serum albumin and α-1-acid glycoprotein. J. Biomol. Struct. Dyn..

[55] Suresh D.V., Mahesha H.G., Rao A.G.A., Srinivasan K. (2007). Binding of bioactive phytochemical piperine with human serum albumin: A spectrofluorometric study. Biopolymers.

[56] Naveenraj S., Anandan S. (2013). Binding of serum albumins with bioactive substances—Nanoparticles to drugs. J. Photochem. Photobiol. C Rev..

