# Supplementary material for: Multi-Spectroscopic and Theoretical Analysis on the Interaction between Human Serum Albumin and a Capsaicin Derivative—RPF101
Source: Biomolecules. 2018 Aug 23;8(3):78. doi: 10.3390/biom8030078 (PMC6164054; doi:10.3390/biom8030078)
Supplement: Supplementary file 1 [file biomolecules-08-00078-s001.pdf]

Supplementary Material

Multi-Spectroscopic and Theoretical Analysis on the Interaction between Human Serum Albumin and a Capsaicin Derivative - RPF101

Otávio Augusto Chaves [1,2], Maurício Temotheo Tavares [3], Micael Rodrigues Cunha [3], Roberto Parise-Filho [3], Carlos Maurício R. Sant'Anna [1] and José Carlos Netto-Ferreira [1,4,*]

1 Institute of Chemistry, Universidade Federal Rural do Rio de Janeiro, BR-465 Km 7, 23970-000 Seropédica-RJ, Brazil; otavioaugustochaves@gmail.com (O.A.C.) ; santana@ufrrj.br (C.M.R.S.)
2 SENAI Innovation Institute for Green Chemistry. Rua Morais e Silva N° 53, Maracanã, 20271030, Rio de Janeiro-RJ, Brazil.
3 Department of Pharmacy, University of São Paulo, Prof. Lineu Prestes Avenue, 580, Bl.13, 05508-900, Butanta, São Paulo-SP, Brazil; mauricio.tavares@usp.br (M.T.T.) ; micaelrc@usp.br (M.R.C.) ; roberto.parise@usp.br (R.P.-F.)
4 National Institute of Metrology, Quality and Technology, 25250-020, Duque de Caxias-RJ, Brazil.
* Correspondence: jcnetto.ufrrj@gmail.com; Tel.: +55-21-96498-4628

Index

1. UV-Vis measurements p.2

Figure S1. UV-Vis spectra for HSA and **RPF101** in PBS solution. [HSA] = 1.00 × 10^{-5} M and [**RPF101**] = 1.32 × 10^{-5} M. p.2

2. Methanol effect on HSA fluorescence and structure p.3

Figure S2. (A) Steady-state fluorescence emission spectra for HSA without and in the presence of 40 μL of methanol at 310 K. (B) CD spectra for HSA without and in the presence of 40 μL of methanol at 310 K. p.4

3. UV-Vis and steady-state fluorescence overlap p.5

Figure S3. Overlap between UV-Vis spectrum of **RPF101** and steady-state fluorescence emission spectrum of HSA at 310 K. [HSA] = [**RPF101**] = 1.00 × 10^{-5} M. p.5

1. UV-Vis measurements

UV-Vis spectra were measured on a Jasco J-815 spectrophotometer in a quartz cell (1 cm optical path). The UV-Vis spectrum for HSA solution (3.0 mL) was carried out in the same concentration of protein used in the steady-state fluorescence measurement (1.00×10^{-5} M). On the other hand, in a 3.0 mL solution of PBS (pH = 7.4) was added the maximum aliquot of **RPF101** used in the steady-state fluorescence measurement (1.32×10^{-5} M) at room temperature (*ca* 298 K) – Figure S1.

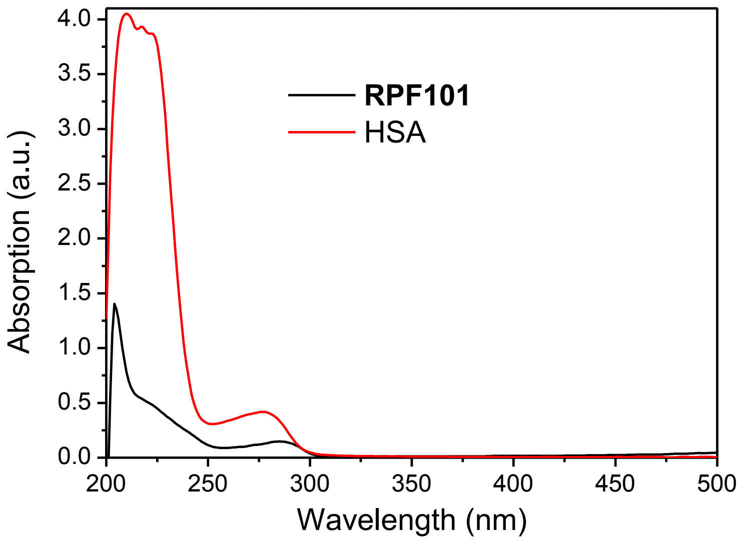

Figure S1. UV-Vis spectra for HSA and **RPF101** in PBS solution. [HSA] = 1.00×10^{-5} M and [**RPF101**] = 1.32×10^{-5} M.

2. Methanol effect on HSA fluorescence and structure

In order to evaluate the methanol effect (solvent used to prepare **RPF101** solution) on the HSA fluorescence and structure, it was conducted steady-state fluorescence and CD experimentsfor HSA without and in the presence of methanol. Steady-state fluorescence and CD were measured on a Jasco J-815 spectrophotometer in a quartz cell (1 cm optical path), employing a thermostated cuvette holder Jasco PFD-425S15F. The steady-state fluorescence spectra were measured in the 290–450 nm range, at 310 K, with λ_{exc} = 280 nm. To a 3.0 mL solution containing an appropriate concentration of HSA (1.00×10^{-5} M in PBS solution), it was added manually 40 μL of methanol (maximum solvent aliquot used in the addition of **RPF101**). On the other hand, CD spectra were measured in the 200–250 nm range, at 310 K. Firstly, the spectrum of a free HSA solution (1.00×10^{-6} M in PBS solution) was recorded and then the spectrum resulting from the addition of the maximum methanol used in the steady-state fluorescence experiments to the HSA solution was also recorded.

As can be seen in the Figure S2, the steady-state fluorescence emission and CD spectra of HSA did not change significantly in the presence of methanol, being clear evidence that the presence of this solvent (40 μL) does not perturb the data obtained in the HSA:**RPF101** studies.

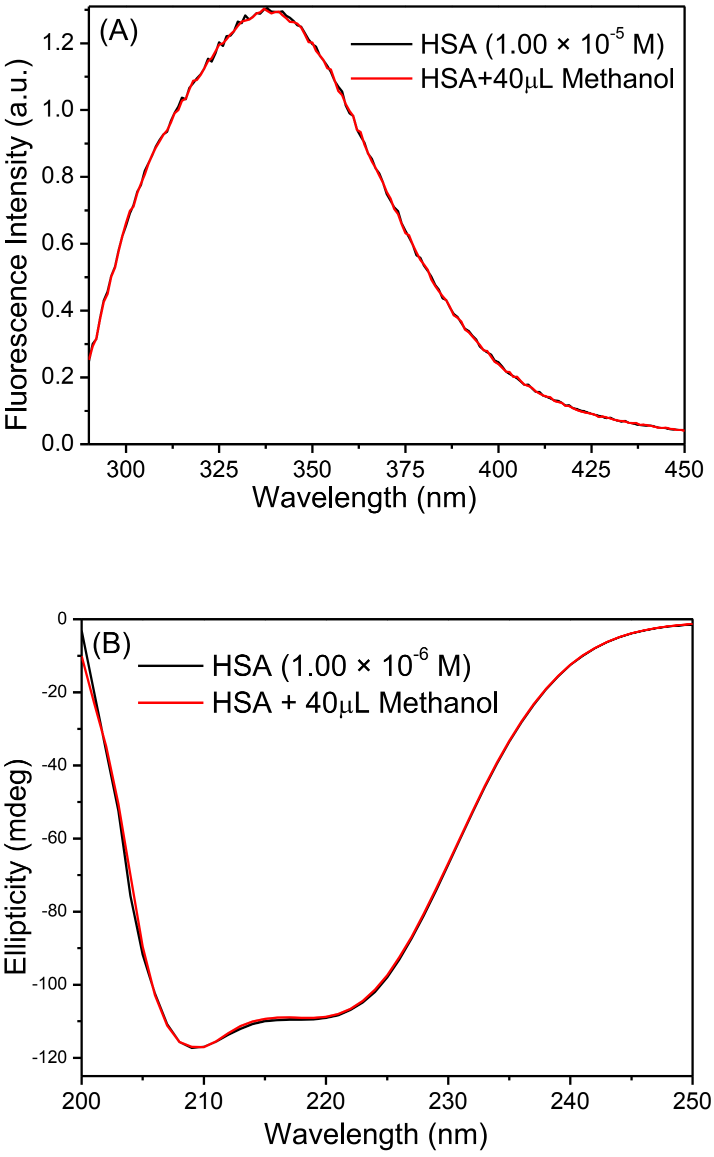

Figure S2. (A) Steady-state fluorescence emission spectra for HSA without and in the presence of 40 μL of methanol at 310 K. (B) CD spectra for HSA without and in the presence of 40 μL of methanol at 310 K.

3. UV-Vis and steady-state fluorescence overlap

UV-Vis and steady-state fluorescence spectra— for **RPF101** and HSA, respectively— were carried out on a Jasco J-815 spectrophotometer in a quartz cell (1 cm optical path), employing a thermostated cuvette holder Jasco PFD-425S15F. The steady-state fluorescence spectrum for HSA (3.0 mL, 1.00 × 10^{-5} M in PBS

solution) was measured in the 290–450 nm range, at 310 K, with λ_{exc} = 280 nm. UV-Vis spectrum for **RPF101** (3.0 mL, 1.00 × 10^{-5} M in PBS solution) was measured in the 290–450 nm range, at 310 K. The overlap between them is represented in the Figure 3S.

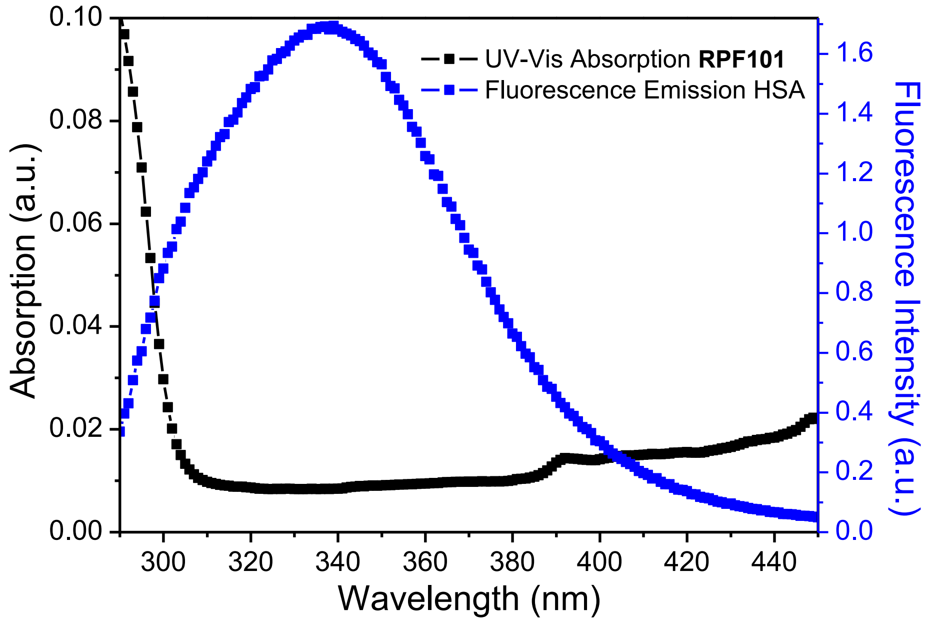

Figure S3. Overlap between UV-Vis spectrum of **RPF101** and steady-state fluorescence emission spectrum of HSA at 310 K. [HSA] = [**RPF101**] = 1.00 × 10^{-5} M.
